# The Endocytic Receptor DEC205 Is Expressed by Brain Endothelial Cells and Is Involved in Regulating the Blood–Brain Barrier

**DOI:** 10.3390/cells15100882

**Published:** 2026-05-12

**Authors:** Sonali Singh, Sabine Ring, Xuan Lei, Mohamad Alabdullah, Yutaka Inaba, Alexander Enk, Karsten Mahnke

**Affiliations:** 1 Department of Dermatology, University Hospital Heidelberg, Im Neuenheimer Feld 440, 69120 Heidelberg, Germany; sonali.singh@med.uni-heidelberg.de (S.S.); sabine.ring@med.uni-heidelberg.de (S.R.); xuan.lei@med.uni-heidelberg.de (X.L.); mohamad.alabdullah@med.uni-heidelberg.de (M.A.); alexander.enk@med.uni-heidelberg.de (A.E.); 2 Department of Dermatology, Wakayama Medical University, 811-1 Kimiidera, Wakayama 641-0012, Japan; ptfjk298@wakayama-med.ac.jp

**Keywords:** DEC205, endothelial cells, blood–brain barrier

## Abstract

**Highlights:**

1. For the first time, we demonstrate the expression of DEC205 by non-immune cells, i.e., brain endothelial cells.
2. In endothelial cells, DEC205 is involved in regulating the tightness of endothelial junctions.

**Abstract:**

The surface receptor DEC205 (alias CD205, NLDC-145, Ly75) has long been a marker for murine dendritic cells and Langerhans cells and functions as receptor to facilitate antigen uptake. We revisited the tissue expression pattern of DEC205 in mice and revealed expression by endothelial cells (ECs) in the central nervous system. Using the murine brain-derived EC line bEnd.3, we could show that DEC205 serves as an endocytic receptor that directs potential ligands to lysosomal compartments. As for its function in ECs, upon engagement by anti-DEC205 antibodies, DEC205 seems to guide Claudin 5, a component of endothelial junctions, away from the surface of ECs to a LAMP-1^+^, degradative compartment. This opens endothelial junctions, eventually allowing leukocytes to migrate from blood to tissue sites. In aggregate, by regulating the blood–brain barrier, DEC205 in ECs may contribute to the regulation of inflammatory processes in the central nervous system.

## 1. Introduction

The molecule DEC205 was originally detected using the NLDC-145 antibody, and its initially unique expression by dendritic cells (DCs) made it a well-accepted marker for DCs [1,2,3]. According to its sequence [4,5], DEC205 (also termed CD205 or Ly75) belongs to the type I C-type lectin-like receptor family. Its closest relatives are the mannose receptor and the phospholipase A2 receptor. All members of this family possess carbohydrate recognition domains (CRDs), a fibronectin type II domain in the extracellular region, and a coated pit motif in their cytoplasmic tail. The latter enables these receptors to mediate endocytosis [6].

The outward-facing CRDs are thought to bind sugar residues in proteins and microorganisms [4,5]. Given that these receptors contain 8–10 different CRDs, they may bind a variety of ligands. However, no defined ligand for the DEC205 receptor has been definitively identified to date. Anecdotal evidence, however, suggests that DEC205 may bind to Cytosine-phosphate-Guanine (CpG), apoptotic cells, and/or keratins [7,8,9]. Without natural ligands, anti-DEC205 (αDEC205) antibodies (Abs) with directly attached proteins were used as surrogate ligands to test the function of DEC205. In DCs, Abs were endocytosed and transported to LAMP-1^+^/MHC class II^+^ compartments (MIICs), where peptides are loaded onto MHC class II molecules and subsequently presented on the cell surface as MHC class II–peptide complexes [10]. From MIICs, DEC205 can recycle back to the cell surface, enabling additional rounds of ligand uptake. This efficient recycling ensures that antigens taken up by DEC205 are better presented by DCs compared to antigens taken up via pinocytosis or phagocytosis. This led to the development of different targeting strategies, using αDEC205 Abs to load DCs with antigens [11,12,13,14,15,16,17]. Initial experiments used model antigens, such as Ovalbumin, coupled to αDEC205 Abs to load DCs in vitro [16], and later known tumor antigens as well as self-antigens were generated and tested for DC-targeting in vivo [11,12,18,19,20].

While DEC205 is strongly expressed by DCs, early immunohistological studies demonstrated staining outside from immune cells, i.e., staining of epithelial cells in the gut, the thymus and the lung as well as of stroma cells in the bone marrow. Among leukocytes, recent studies have also detected expression in B cells [21] and neutrophilic granulocytes [22]. Since both of these cell types express MHC class II molecules and can present antigens, a functional role for DEC205 as an antigen-capture receptor is plausible. Re-evaluation of the expression of DEC205 in different tissues of mice has revealed that its expression is not restricted to leukocytes. Our data indicate that endothelial cells (ECs) in the central nervous system (CNS) specifically bind αDEC205 Abs. Regarding the function of DEC205 in ECs, we show that engagement of DEC205 by Ab targeting affects the regulation of tight junctions. In particular, DEC205 regulates the surface expression of Claudin 5, a protein involved in tightening endothelial junctions, by mediating its endocytosis and degradation in lysosomal compartments. In summary, in ECs, DEC205 plays an important role in regulating tight junctions through controlled endocytosis of tight junctional proteins and in stabilizing the blood–brain barrier.

## 2. Materials and Methods

### 2.1. Cells and Mice

bEnd.3 cells [23] were purchased from LGC Standards GmbH, Wesel, Germany, and cultivated in a complete medium consisting of RPMI 1640, 10% FBS and 1% PenStrep (all from Bio&Sell, Feucht, Germany). DEC205^−/−^ mice were purchased from The Jackson Laboratory (Bar Harbor, ME, USA) and C57BL/6N mice were purchased from Janvier Labs (Le Genest-Saint-Isle, France). Animals were bred in the central animal facility of the University of Heidelberg in accordance with the guideline for animal welfare.

### 2.2. Antibodies and Chemicals

αDEC205 Abs (clone NLDC-145) and respective isotype controls (αGalactosidase; clone GL117) were isolated from hybridoma supernatants according to standard procedures using the ÄKTA pure^TM^ chromatography system (Cytiva Europe GmbH, Freiburg, Germany). For experiments using flow cytometry, the following Abs were purchased from BioLegend, Koblenz, Germany: αDEC205-PE (clone NLDC145), αCD31-AF488, αCD144-APC, αCD54-BV421 and αCD45-PE/Cy7. αCD62P-BV510 was obtained from BD, Becton Dickinson GmbH, Heidelberg, Germany. The DEC205 clone 205YEKTA was purchased from Thermo Fisher Scientific, Dreieich, Germany. Individual sufficient concentrations of all Abs were titrated in pre-experiments. For microscopic analysis and endocytosis experiments, the following additional Abs were used: αCD11b-AF488, αMHCII-AF488, αCD144-FITC, αRatIgG2a-PE, αCD71-PE (all from BioLegend) and LysoTracker DND-26 from Thermo Fisher Scientific, Dreieich, Germany. Nuclei were counterstained either with DAPI (4,6-Diamidino-2-Phenylindole) or with Hoechst 33342, both from Thermo Fisher Scientific. Cryosections from frozen tissues were stained with purified rabbit-anti-mouse CD31, rat-anti-mouse Lamp1 or rabbit-anti-mouse Claudin 5 Abs (all from Thermo Fisher Scientific) followed by the respective secondary antibodies’ goat-anti-rabbit AF488 or goat-anti-rat TRITC from BIOZOL, Hamburg, Germany.

Recombinant mouse TNFα and IFNγ were obtained from Miltenyi Biotec, Bergisch Gladbach, Germany. CCL21 was obtained from Biolegend, Koblenz, Germany. FITC-labeled ODN1668 was purchased from MCE (Hycultec, Beutelsbach, Germany).

### 2.3. Preparation of Single-Cell Suspension and Flow Cytometry

To prepare single-cell suspensions from bEnd.3 monolayers, the cells were first detached from the bottom of the 75 cm^2^ culture flasks with 3 mL Trypsin/EDTA for 5 min at 37 °C, 5% CO_2_. After stopping the reaction with 10 mL complete medium, cells were counted and washed in FACS buffer (PBS + 3% FBS). Surface molecules were stained with the respective fluorescently labeled Abs for 30 min at 4 °C. All antibodies have been titrated before and were used as following concentrations (all as µg/10^6^ cells/100 µL FACS-buffer): 0.01 µg αDEC205-PE, 0.5 µg αCD31-AF488, 0.05 µg αCD144-APC, 0.1 µg αCD54-BV421, 0.1 µg αCD45-PE/Cy7 and 0.1 µg αCD62P-BV510. After another washing step with FACS buffer, stained cells were analyzed by a Gallios Flow Cytometer (Beckmann Coulter GmbH, Krefeld, Germany) and multicolor flow cytometry data were analyzed using Kaluza 2.1 software. For detection of intracellular DEC205, cells were first stained for surface markers as described above, fixed and permeabilized using an intracellular Fixation and Permeabilization kit (Thermo Fisher Scientific) and stained with *α*DEC205-PE (0.01 µg/10^6^ cells/100 µL) for 1 h at 4 °C. Flow cytometry data were acquired as described before. Stained single-cell suspensions were additionally microscopically analyzed using the EVOS M7000 cell imaging system (Thermo Fisher Scientific).

### 2.4. Live Cell Imaging

bEnd.3 monolayers were grown in 4 chamber µ-Slides (Ibidi, Gräfeling, Germany). Therefore, 1 × 10^4^ cells were seeded in each well and incubated at 37 °C, 5% CO_2_ for 24 h. The dense bEND.3 monolayers were then stained with αCD31-AF488 along with αDEC205-PE Abs (both 1 µg/mL). WT and DEC205^−/−^ bone marrow-derived dendritic cells (BMDCs) were stained with αMHCII-AF488 (1 µg/mL) together with αDEC205-PE (1 µg/mL) Abs serving as positive (WT) and negative (CD205^−/−^) control for these experiments. As indicated in the figure legends, nuclei were counterstained using Hoechst 33342. Images were acquired using a Nikon confocal fluorescence microscope (Nikon and Crest optics, Düsseldorf, Germany) with a Cicero spinning disk device. Whenever appropriate, pictures were simultaneously taken using phase contrast, and overlays of fluorescence and brightfield images are displayed as indicated in the legends. For endocytosis experiments, dense monolayers of the bEND.3 cells were first treated with αDEC205-PE together with αCD144-AF488 Abs (both 1 µg/mL) for 30 min at 37 °C. Thereafter a low-pH acid wash (0.5% acetic acid in 0.5 M NaCl) for 45 s was used to remove remaining surface Abs. Cells were washed and nuclei were counterstained with Hoechst 33342. Images were captured before and after the acid wash using the confocal fluorescence microscope.

For other experiments, monolayers of bEND.3 cells in the 4 chamber µ-Slides were activated with 10 ng/mL IFNγ together with 10 ng/mL TNFα for 4 h at 37 °C, 5% CO_2_. In some chambers, cells were additionally treated with either αDEC205 or control αGL117 (both 1 µg/mL) for 2 h. After cells were washed, they were fixed with 4% PFA, blocked and stained with Abs against Lamp-1 and Claudin 5 (both 2 µg/mL) followed by the respective secondary Abs (both 2 µg/mL) and images were taken using the confocal fluorescence microscope.

### 2.5. Intracellular Trafficking of Endocytosed αDEC205 Abs

Monolayers of bEnd.3 cells in 4 chamber µ-Slides were either treated with 1 µg/mL transferrin receptor αCD71-PE or with αDEC205-PE at the same concentration. Both groups were additionally incubated with 50 nM LysoTrackerGreen DND-26 for 15 min or 30 min at 37 °C, 5% CO_2_. After three washes, the nuclear dye Hoechst 33342 was added and images were taken using an inverted spinning disk confocal microscope as cited before.

### 2.6. Cryosections and Staining

WT (C57BL/6N) and DEC205^−/−^ mice were euthanized and tissues such as brain, eye, spinal cord, ear, lymph nodes, lung and testis were embedded in OCT Tissue-Tek (Sakura Finetek Europe, Umkirch, Germany). OCT-embedded tissues were snap-frozen in liquid nitrogen. Tissue samples were sectioned using a cryostat (Leica Microsystems, Wetzlar, Germany) to a thickness of 5 μm. Cryosections were collected on slides, incubated overnight at 4 °C and successively air-dried. After fixation with 4% PFA (10 min at RT), sections were blocked and permeabilized with 3% goat serum in PBS and 0.1% Triton X-100 for 30 min and finally incubated with the appropriate primary Abs αCD31 (3 µg/mL) and αCD205 (1 µg/mL) in PBS, 1% BSA for 1 h at RT. After several washings, sections were incubated for another hour with the fluorescently labeled respective secondary Abs goat-anti-rabbit AF488 and goat-anti-rat TRITC (both 2 µg/mL) followed by counterstaining with DAPI for 2 min.

### 2.7. Targeting ECs in the CNS by Intravenous Injection of αDEC205

The monoclonal rat Abs αDEC205 (NLDC-145), αGL117 (isotype control), or αCD31 (Bio X Cell, Rüsselsheim, Germany) were injected intravenously (i.v.) into the tail vein of C57BL/6N and DEC205^−/−^ mice. Each mouse received 10 µg of the respective antibody diluted in 100 µL of sterile PBS. Within 10 min, mice were euthanized and brains, ears, lymph nodes, and spinal cords were snap-frozen in liquid nitrogen. Cryosections (5 μm) of the different tissues were prepared and stained with a TRITC-labeled goat anti-rat secondary Abs (2 µg/mL) to detect the in vivo bound i.v. injected primary Abs. In some experiments, additional staining with αCD31-AF488 was performed to visualize the ECs. The nuclei were stained by DAPI.

### 2.8. Transmigration Experiments Using Boyden Chambers

The endothelial monolayer was prepared by culturing bEnd.3 cells (1 × 10^5^ cells per transwell) on 3 μm pore-sized 6-well transwell inserts at 37 °C, 5% CO_2_ for 2 days. The transendothelial electrical resistance (TEER) was measured using an EVOM3 epithelial volt/Ohm meter (World Precision Instruments, Friedberg, Germany). TEER values of 70–80 Ω/cm^2^ were used to ensure integrity of the bEnd.3 monolayers.

The endothelial monolayers were either left untreated (no activation) or activated with IFN-γ (10 ng/mL) and TNF-α (10 ng/mL) for 4 h. Thereafter monolayers were either treated with αDEC205 (1 µg/mL) or αRatIgG (1 µg/mL) for up to 6 h. At 0, 1, 2, 4 and 6 h after treatment with Abs, TEER was measured across the differently treated cellular monolayers using the EVOM3 epithelial volt/Ohm meter (World Precision Instruments, Friedberg, Germany).

For the transendothelial migration assay of leukocytes the endothelial monolayers of bEND.3 cells were prepared on the transwell inserts like described above. The monolayers were either activated with IFN-γ (10 ng/mL) and TNF-α (10 ng/mL) for 4 h before treatment with αDEC205 (1 µg/mL) or αRatIgG (1 µg/mL) for additional 4 h occurred. Other experimental settings were not activated before the treatment with αDEC205 or αRatIgG. One monolayer was not treated or activated at all, serving as the control. CD4^+^ T cells were isolated from spleens of naïve C57BL/6N mice using a CD4^+^ T cell Isolation Kit from Miltenyi Biotec, Bergisch Gladbach, Germany. Next, 1 × 10^5^ CD4^+^ T cells/2.5 mL complete medium were pipetted into the upper chamber. The lower chambers were filled with 3 mL complete medium containing recombinant purified CCL21 (400 ng/mL) as chemoattractant. The Boyden chambers were incubated for 4 h at 37 °C and 5% CO_2_. After incubation, the migrated CD4^+^ T cells in the lower chambers were visualized using microscopy, and their numbers were quantified using Celleste software, which calculated the number of transmigrated cells per area based on a selected size of cells.

### 2.9. mRNA Expression and qRT-PCR

RNA from bEND.3 cells and neutrophils isolated from bone marrow of WT and CD205^−/−^ mice were extracted using the RNeasy Plus Mini Kit (Qiagen, Hilden, Germany) based on the manufacturer’s protocol. Total RNA concentration and purity were determined by measuring the absorbance at 260 nm and 260/280 nm and 260/230 nm ratios, respectively (Denovix, Wilmington, DE, USA). RNA was reverse-transcribed to yield cDNA by the RevertAid First Strand cDNA Kit according to standard protocols. Real-time RT-PCR was performed using the PowerUp™ SYBR Green Master Mix and the StepOnePlus Real-Time PCR system. For detection of DEC205 expression the following gene-specific primers for exon 1 and exon 2 were used: Ly75 FP1: 5′-ACATGACCCATGACGCAATATAA-3′, Ly75 RP1: 5′-TCGGAATGAGGAGGAGAATACCT-3′, Ly75 FP2: 5′-GACCCA TGAGGCAATATAATTGAAG-3′ and Ly75 RP2: 5′-TCGGAATGACGAGCAGAATAG CTT-3′ (Eurofins, München, Germany). Gene expression was quantified using the ΔΔCT method (Livak and Schmittgen, 2001) and normalized to GAPDH expression.

For analyzing the multiple genes involved in endothelial junctions of bEND.3, qRT-PCRs using the mouse adherin-tight junction primer library (MATJ-I) (Biomol, Hamburg, Germany) were performed. Therefore bEnd.3 monolayers were activated with IFN-γ (10 ng/mL) together with TNF-α (10 ng/mL) for 4 h. Different groups were either treated with αDEC205 or αRatIgG as control for additionally 2 h. mRNA was extracted and reverse-transcribed as outlined above. cDNA was subjected to qRT-PCRs using the MATJ-I specific primer library and quantified by the ΔΔCT method with β2-microglobulin as the reference gene.

## 3. Results

### 3.1. The Endothelial Cell Line bEnd.3 Expresses DEC205

**Figure 1:**
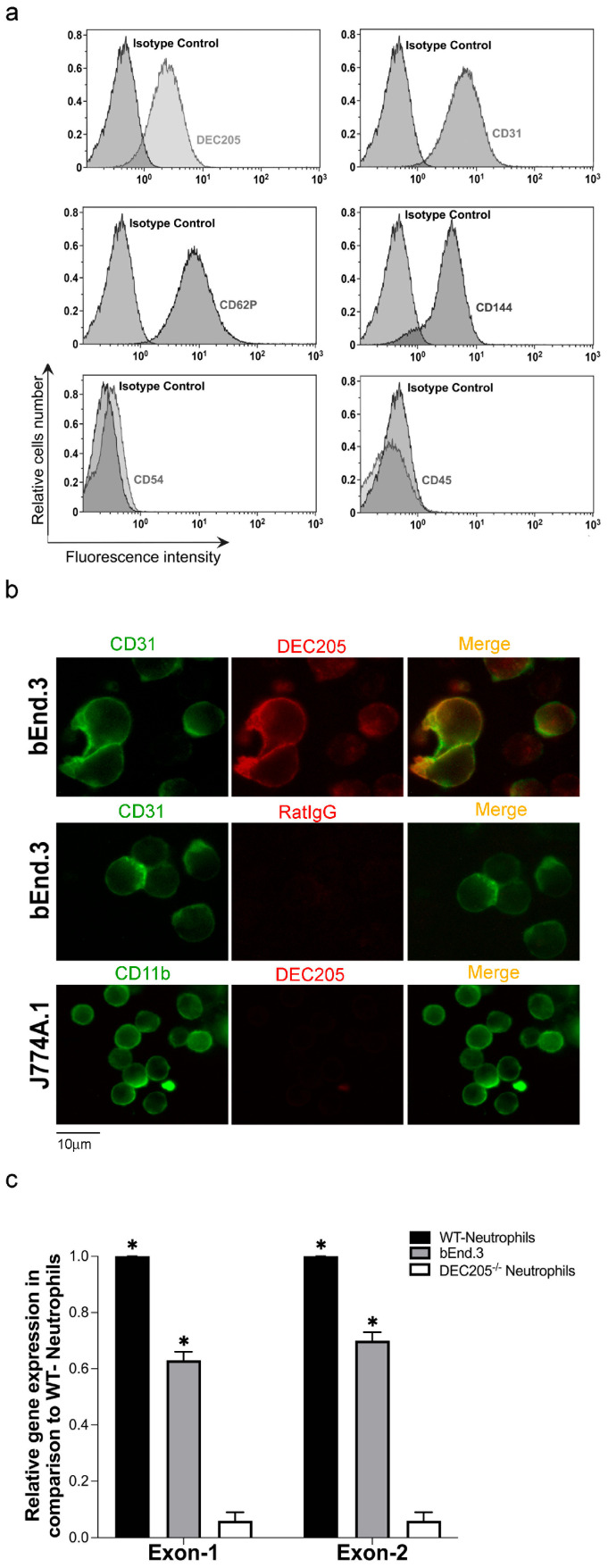
DEC205 is expressed by bEnd.3 cells. (**a**) Single-cell suspensions of bEnd.3 cells were stained with fluorescently labeled Abs as indicated and analyzed by FACS. Histograms depict the mean fluorescence intensity of the stained surface markers and the suitable isotype controls. (**b**) bEnd.3 cells and the monocytic cell line J774A.1 were stained in suspension with fluorescently labeled Abs as indicated. Images were captured by fluorescence microscopy. (**c**) mRNA was isolated from bEnd.3 cells. mRNA from neutrophils isolated from bone marrow of WT and DEC205^−/−^ mice served as positive and negative control. qRT-PCR was performed using specific DEC205 exon-1 (Ly75.1) and exon-2 (Ly75.2) primers. GAPDH expression served as housekeeping control. Expression relative to neutrophiles isolated from WT mice is shown. * Marks a significant difference to DEC205^−/−^ cells (*t*-test *p* ≤ 0.05).

To assess whether DEC205 is expressed by ECs, the murine brain EC line bEnd.3 was analyzed by flow cytometry. The cells were stained with αDEC205 Abs and with Abs against other surface markers specific for ECs, followed by FACS analysis. Figure 1a demonstrates that bEnd.3 cells highly express DEC205 in comparison to staining with isotype control Abs. Other EC-specific markers, such as CD31, CD144, and CD62P, were also expressed like expected, whereas the lymphocyte marker CD45 was negative in these cells, and the adhesion molecule CD54 was only weakly expressed in the unstimulated, naïve bEnd.3 cells. In further experiments, single-cell suspensions of cells as indicated were stained in suspension with αDEC205, αCD31 and αCD11b Abs, and analyzed by immunofluorescence microscopy. These results confirmed expression of DEC205 by bEnd.3 cells again (Figure 1b), whereas the macrophage cell line J774A.1 showed no binding of αDEC205, but as expected, expressed the monocyte-specific marker CD11b. Finally, primers targeting exon 1 and exon 2 of DEC205 were designed, and qRT-PCR was performed. The PCR data shown in Figure 1c reveal strong expression of DEC205 in bEnd.3 cells. This expression level was comparable to that observed in neutrophils isolated from bone marrow of WT mice, which are known to be highly positive for DEC205 [22], whereas neutrophils isolated from DEC205^−/−^ mice showed no detectable expression. On the basis of these data, we can assert that the murine brain-derived EC line bEnd.3 is positive for the endocytic receptor DEC205.

### 3.2. DEC205 in bEnd.3 Cells Mediates Endocytosis of Surface-Bound Antibodies

**Figure 2:**
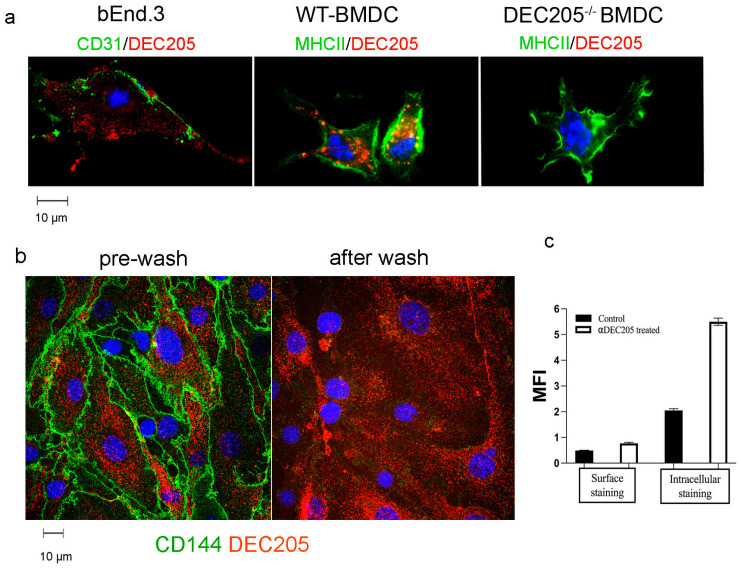
DEC205 facilitates receptor-mediated endocytosis in bEnd.3 cells. (**a**) bEnd.3 cells and BMDCs generated from WT or DEC205^−/−^ mice were incubated with αDEC205 antibodies for 30 min. After fixation and permeabilization, cells were stained with secondary Ab goat-anti-rat TRITC and with αCD31-AF488 or αMHCII-AF488. Nuclei were counterstained with DAPI and are depicted in blue. Images were taken by fluorescence microscopy. (**b**) Monolayers of bEnd.3 cells were incubated with αDEC205-PE and αCD144-FITC for 30 min followed by a low-pH wash, to remove surface-bound Abs. Live-cell imaging before and after acidic wash analyzed presence of intra- and extracellular antibodies. Nuclei stained with Hoechst are depicted in blue. (**c**) bEnd.3 cells were pulsed with αDEC205 or αRatIgG (control) for 15 min and chased for 30 min. Thereafter, single-cell suspensions were stained with secondary Abs with or without prior permeabilization. The data show the mean fluorescence intensities (MFIs) as analyzed by FACS.

As DEC205 has been shown to function as an antigen uptake receptor in DCs, uptake of surrogate ligands, i.e., surface-bound αDEC205 Abs by bEnd.3 and BMDCs, was analyzed next. After incubating bEnd.3 and BMDCs with αDEC205 Abs, cells were stained with Tetramethylrhodamine isothiocyanat (TRITC)-labeled secondary reagents and AF488 labeled αMHCII or αCD31 Abs respectively (Figure 2a). In bEnd.3 cells, staining with αCD31 Abs revealed lining of the outer cell membrane, and for the most part DEC205 was detected intracellularly. Similarly, in BMDCs uptake of surface-bound αDEC205 Abs and accumulation in MIICs could be detected, whereas BMDCs derived from DEC205^−/−^ mice were devoid of any staining with αDEC205 Abs. To test whether uptake of αDEC abs results in antigen presentation, as described for DCs, bEnd.3 cells, pulsed with DEC205:OVA conjugates [16], were set up with Ovalbumin-specific DO11.10 CD4^+^ T cells. However, no significant proliferation could be recorded (Supplemental Figure S1).

To verify that αDEC205 Abs were endocytosed by ECs after 30 min, bEnd.3 cells were incubated with PE-labeled αDEC205 Abs and FITC-labeled αCD144 Abs, binding to the vascular endothelial cadherin for 30 min. Microscopic images were taken, followed by a wash with acidic buffer (pH 5.5) to remove all surface-bound Abs. After a short recovery time in the incubator (5 min), cells were imaged again [24]. As shown in Figure 2b, Abs bound to the surface molecule CD144 were no longer detectable after acid wash, indicating a dissociation of the Abs from the surface. In contrast, αDEC205 Abs were still detectable intracellularly after acid treatment, suggesting uptake and intracellular localization of αDEC205 Abs in bEnd.3 cells. These results are further backed by confocal images and 3-dimensional rendering. Here, internal accumulation of αDEC205 abs in the perinuclear area was recorded (Supplemental Figure S2).

Likewise, experiments using FACS analysis of fixed vs. fixed and permeabilized bEnd.3 cells (Figure 2c) show similar results [25], i.e., intracellular staining yielded a higher mean fluorescence of DEC205 as compared to surface staining alone, confirming our notion that αDEC205 Abs are taken up after binding to the DEC205 receptor and are stored intracellularly.

**Figure 3:**
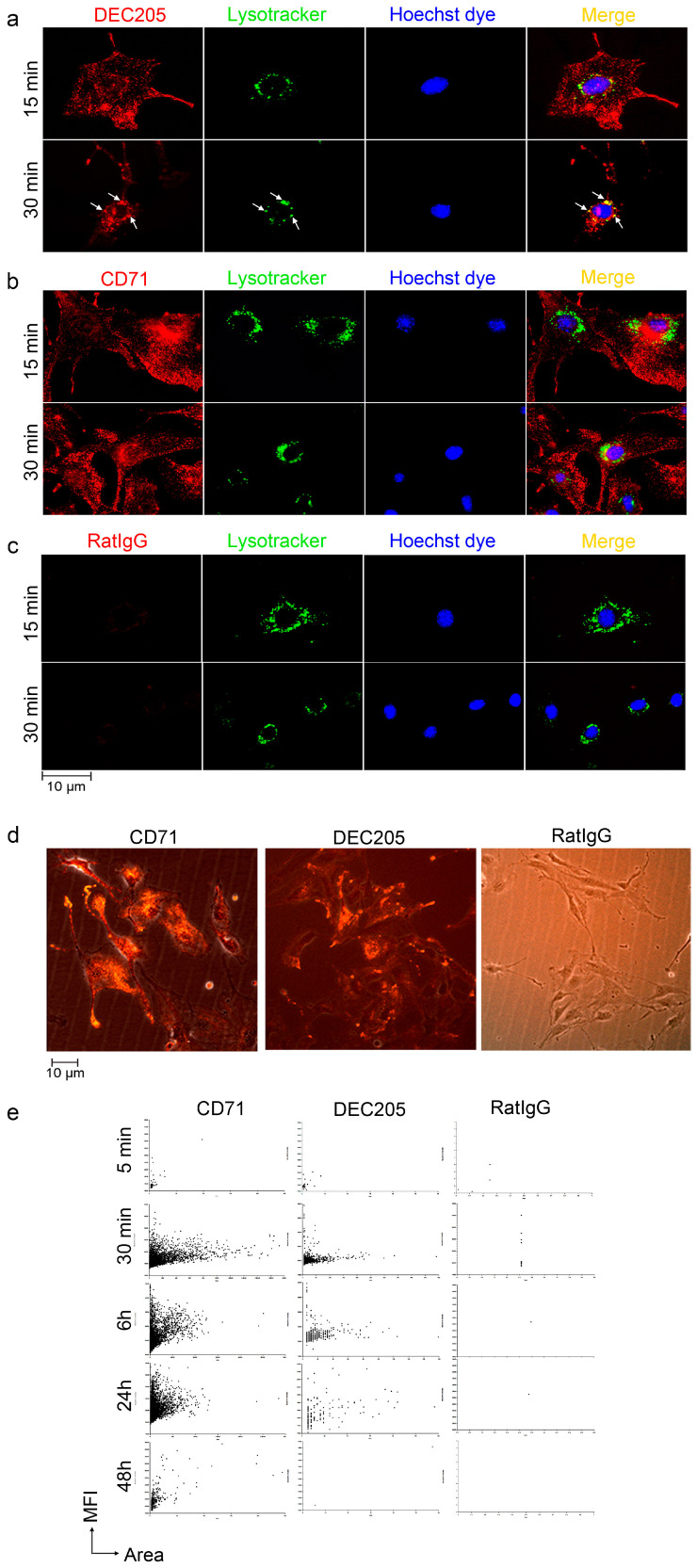
DEC205 targets to lysosomal compartments. bEnd.3 cells were treated with (**a**) αDEC205-PE, (**b**) αCD71-PE (transferrin receptor), and (**c**) αRatIgG2a Abs, along with LysoTracker Green for 15 min and 30 min. Live cells were imaged using spinning disk microscopy. (**a**) White arrows indicate colocalization of αDEC205-PE with LysoTracker Green. The nuclei were counterstained with Hoechst 33342 and displayed in blue. (**d**) bEnd.3 cells were incubated with either αDEC205-PE, αRatIgG2a-PE, or αCD71-PE for 30 min, live cell images were taken with fluorescence settings for PE and phase contrast, and pictures were overlaid using Nikon software. (**e**) Cells were treated like in (**d**) and whole cell culture dishes were analyzed for fluorescence intensity (MFI) and size of cells (area) at timepoints as indicated. Results are displayed as graph using Gen5 3.15 software.

In a series of experiments, the fate of endocytosed αDEC205 Abs was analyzed in greater detail. Pulse chase experiments with LysoTracker, which is transported and detectable in lysosomal compartments only, and αDEC205 pulsed cells (Figure 3a), revealed targeting of αDEC205-tagged receptors to late endosomal compartments within 30 min. In contrast, the prototypic recycling receptor for transferrin (CD71) [26,27] did not enter LysoTracker-positive compartments, although uptake and presence in endocytic vesicles were clearly detectable (Figure 3b). Here, the vesicles mostly remained in the peripheral cytoplasm. Moreover, we tested whether αDEC205 antibodies derived from a different clone (i.e., Yekta) and a previously described DEC205 ligand (i.e., CpG) are also taken up by bEnd.3 cells. Both were indeed internalized (Supplemental Figure S3); however, fluorescently labeled CpG exhibited a markedly diminished uptake rate compared to αDEC205 antibodies, suggesting that CpG is likely endocytosed via a mechanism independent of DEC205 [28].

Furthermore, the fate of endocytosed αCD71 and αDEC Abs was compared using cellular imaging of tissue culture plates (Figure 3d). Single photomicrographs of culture dishes were taken by immunofluorescence microscopy and by phase contrast and overlaid (Figure 3d). These exemplary pictures revealed that fluorescence of endocytosed αDEC205 and αCD71 antibodies can be detected 30 min after pulsing, with DEC205 being visible as early as 5 min after pulsing (Supplemental Figure S4). In contrast, αRatIgG control abs were not detectable. In an extended timecourse approach, whole plates were automatically analyzed for the total number of cells and their mean fluorescence was assessed (Figure 3e). Scatter plots reveal decay of endocytosed αDEC205 Abs within 24 h after chase, whereas αCD71 abs were detectable in substantial amounts for more than 48 h. Thus, these data confirm our notion of DEC205 receptor targeting to late endosomal compartments and may indicate degradation of ligands endocytosed by DEC205.

### 3.3. ECs in Neuronal Tissues Express DEC205 In Vivo

**Figure 4:**
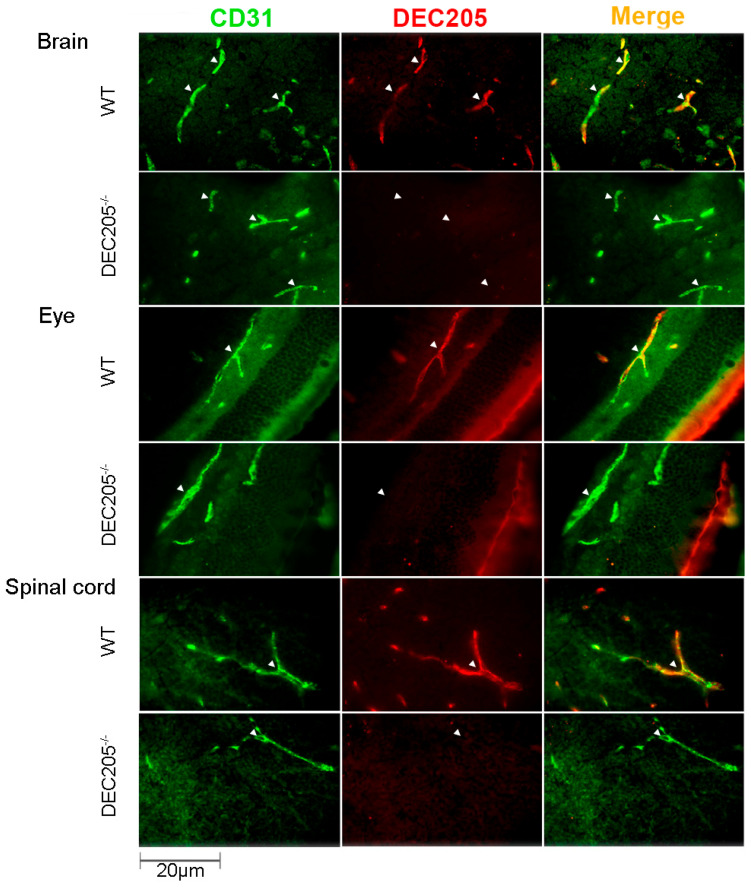
DEC205 is exclusively expressed by endothelial cells in tissues of the CNS. Cryosections of frozen tissues from the brain (cerebellum), eye (retina), and spinal cord (cervical) of C57BL/6N (WT) and DEC205^−/−^ mice were stained with αCD31 together with αDEC205 Abs, followed by the respective secondary Abs. Images were captured by using immunofluorescence microscopy, and arrows indicate exemplary positively stained vessels and cells. Double labeling of ECs is shown in “Merge.”

In order to confirm the expression of DEC205 by ECs in vivo, various tissues were analyzed for DEC205 expression on vascular endothelial cells by immunofluorescence staining. As depicted in Figure 4, ECs, visualized by staining with αCD31, in brain, eye and spinal cord of WT mice, express DEC205, whereas in other organs, comprising lymph nodes, skin, lung, testis, and choroid plexus ECs did not exhibit any binding of αDEC205 Abs (Supplemental Figure S5). In conclusion, DEC205 is expressed by brain ECs in vivo.

**Figure 5:**
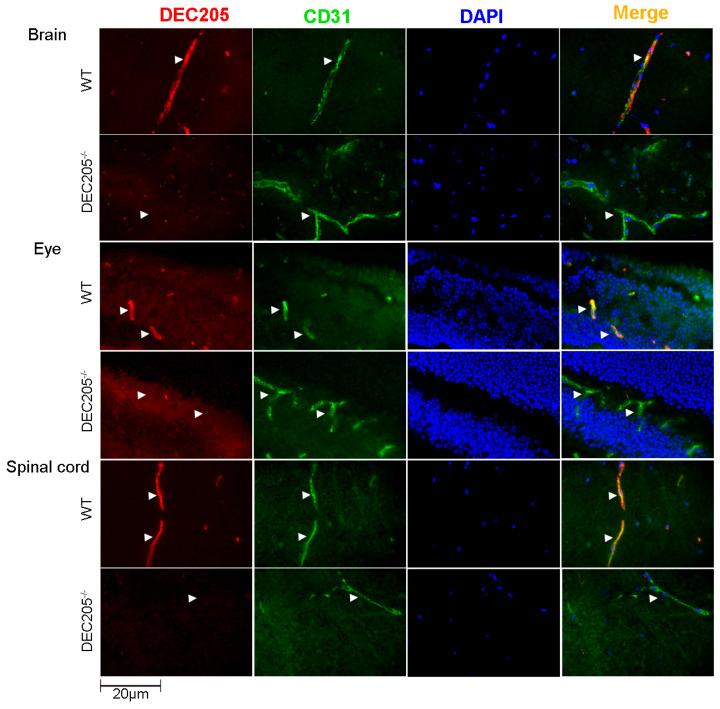
αDEC205 antibodies specifically target endothelial cells in the CNS in vivo. Cryosections of frozen tissues (brains, eyes, and spinal cord) from αDEC205-injected mice were stained with goat-anti-rat-TRITC secondary Abs to visualize the injected primary Abs which have bound in vivo to the vessels (displayed in red). Sections were co-stained with αCD31-AF488, displayed in green. Merged images display colocalization of both markers. Nuclei were counterstained with DAPI, appearing in blue. Arrowheads mark similar areas of the sections indicating double labeling or not.

As Abs against DEC205 strongly bind to ECs in vitro, we next analyzed whether αDEC205 Abs are able to target brain ECs in vivo, as similar approaches have already been successfully been tested for loading DCs in vivo with antigens [11,12,13]. To this end, αDEC205 Abs were i.v. injected into animals, and brains, eyes and spinal cords were snap-frozen, sectioned and stained with TRITC-labeled secondary reagents, for detection of the in vivo bound αDEC205 Abs, together with αCD31-AF488 Abs to mark ECs. As shown in Figure 5, brain, eye and spinal cord isolated from WT mice after injection of αDEC205 Abs showed a strong binding of the Abs to the vascular endothelium in vivo. In contrast, organs isolated from DEC205^−/−^ animals showed no binding of the injected αDEC205 Abs at all, like expected. Analyzing ECs in organs which do not belong to the CNS, like lymph nodes, skin and lungs, no binding of the injected αDEC205 Abs on ECs was detected (Supplemental Figure S6). Thus, these data indicate that αDEC205 Abs specifically bind to vascular ECs of the CNS, upon i.v. injection.

### 3.4. Treatment of ECs with αDEC205 Induces Decreased Transendothelial Electrical Resistance

**Figure 6:**
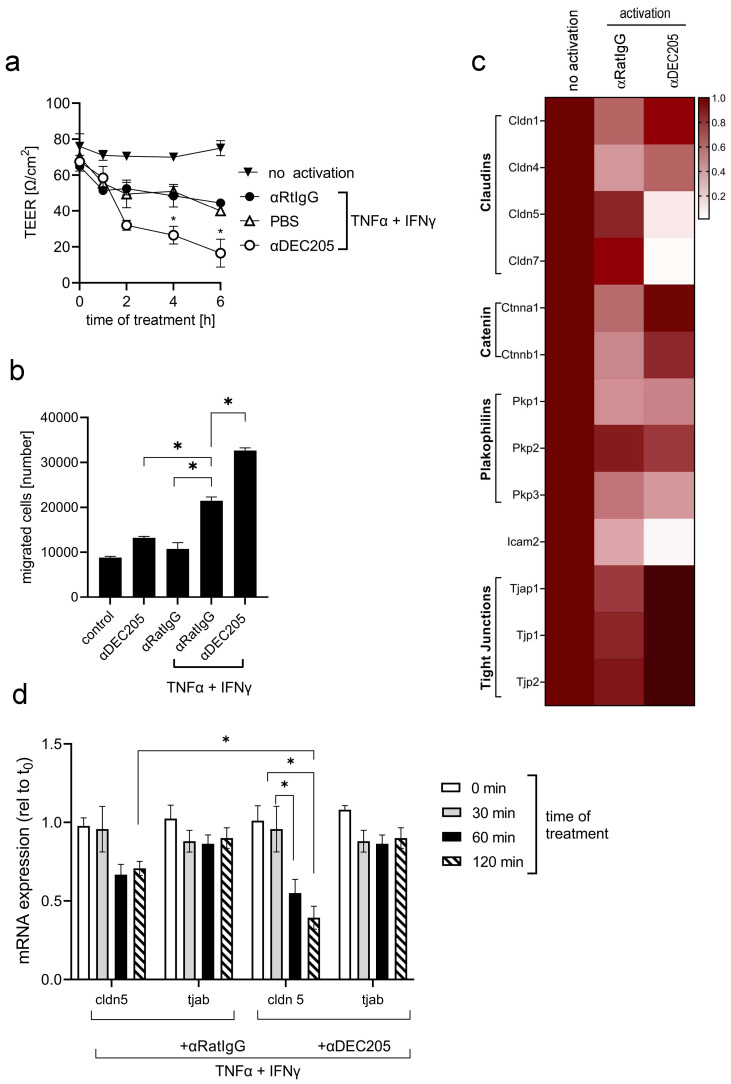
DEC205 regulates endothelial tight junctions. (**a**) Monolayers of bEnd3 cells, grown on the inserts of 6-well Boyden chambers, were activated with IFNγ (10 ng/mL) and TNFα (10 ng/mL) for 4 h before the cells were treated with Abs as indicated; TEER was measured before treatment, 1, 2, 4, 6 h later. The means ± SD are given (n = 3; * is significant to PBS; *t*-test *p* ≤ 0.05). (**b**) Inserts of 6-well Boyden chambers were grown with monolayers of bEND.3 cells and were treated like described in (**a**). Other groups were not activated and only treated with αDEC205 or αRatIgG for 2 h. Next, 1 × 10^5^ isolated CD4^+^ T cells were added to the upper compartment of the Boyden chambers and migrated T cells were counted after 4 h of incubation. The means ± SD are given (n = 3; * is significant; *t*-test *p* ≤ 0.05). (**c**) Monolayers of bEND.3 cells in 75 cm^2^ culture flasks were activated with IFNγ and TNFα for 4 h and treated with either αDEC205 or αRatIgG for additional 2 h. Control group was left untreated at all (no activation). mRNA was extracted and analyzed by qRT-PCR using specific primers as indicated. Gene expression was normalized to β2-microglobulin and compared to the expression levels of untreated endothelial cells, which were set as the baseline (1.0). (**d**) bEND.3 cells were cultured, activated and treated with antibodies like described in (**c**). After indicated timepoints, cells were harvested and mRNAs were prepared. qRT-PCR was performed with tjab1 and cldn5-specific primers and expression was analyzed in comparison to the housekeeping gene β actin, set to 1. * Significant difference to αIgG; *t*-test *p* ≤ 0.05.

As one of the key functions of ECs is to secure the tightness of blood vessels and to regulate the trafficking of leukocytes during inflammation, we next investigated the functional effects of αDEC205 Ab binding to the DEC205 receptor on bEnd.3 cells using Boyden chambers, which allowed us to analyze the transendothelial electrical resistance (TEER) of the differently treated EC monolayers [29]. Therefore, dense monolayers of bEnd.3 cells were either activated or left untreated. Additionally, some of the layers were treated with αDEC205 Abs or respective isotype controls (αGL117) and TEER was measured at different timepoints up to 6 h after treatment. The data in Figure 6a show an expected decrease of TEER in activated monolayers of bEnd.3 cells in the course of the experiment, indicating an opening of EC junctions upon activation by a combination of IFNγ and TNFα. After additional treatment with αDEC205 Abs, TEER dropped even further as compared to isotype (αRatIgG)-treated samples. After removal of the stimulatory reagents and the respective Abs, TEER increased gradually reached its original levels after 8–12 h. In addition, migration assays with Boyden chambers were performed. Isolated CD4^+^ T cells were placed on top of bEnd.3 monolayers grown on semipermeable membranes, activated and treated with Abs as indicated (Figure 6b), and to the lower compartment CCL21 was added. Migrated cells were counted after 4 h. As depicted in Figure 6b, αDEC205 antibody treatment led to increased migration of T cells through activated endothelium, which is in line with data obtained by measuring TEER.

To confirm our hypothesis in vivo, mice were i.v. injected with αDEC205 Abs and respective isotype control Abs, followed by Evans Blue injection. Mice were euthanized and brains were prepared 30 min thereafter (Supplemental Figure S7). Here, a stronger extravasation of Evans Blue was detected in WT mice injected with αDEC205 Abs as compared to isotype-injected controls. Moreover, in DEC205-deficient animals, injection of αDEC205 Abs failed to induce increased Evans Blue extravasation. Thus, these data indicate that triggering the endocytic receptor DEC205 on ECs by specific Abs opened the endothelial junctions.

### 3.5. αDEC Treatment Regulates the Expression of Junctional Proteins in ECs

**Figure 7:**
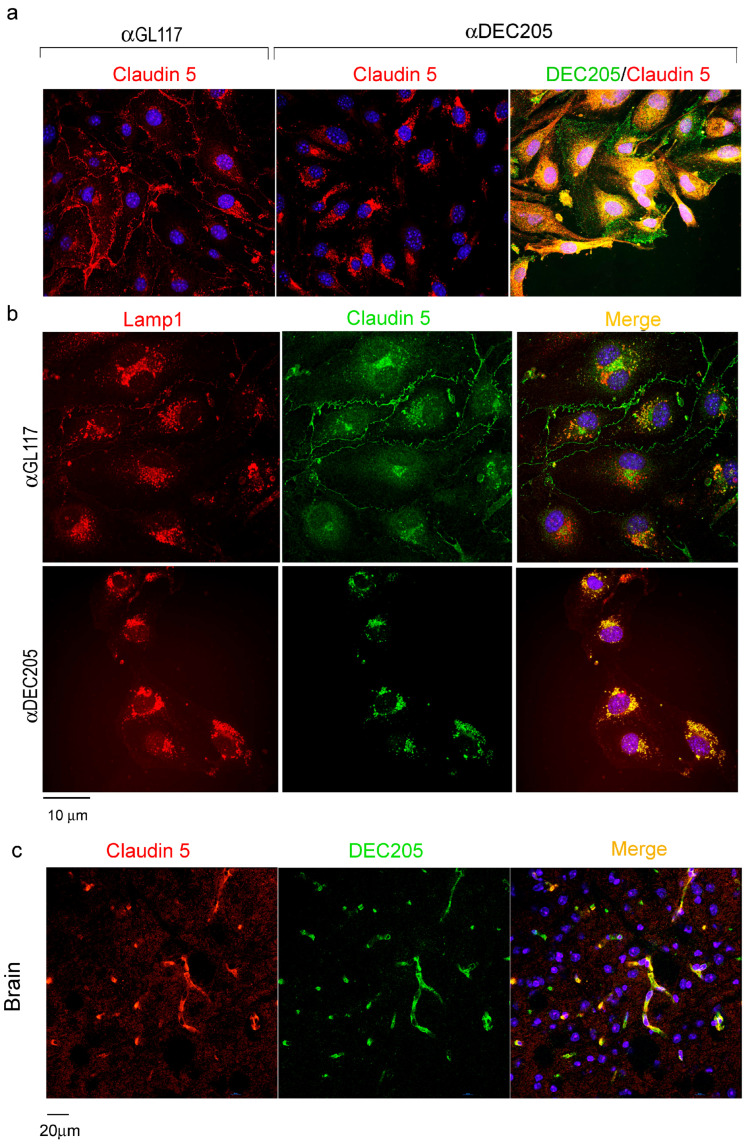
DEC205 and Claudin 5 colocalize in ECs of murine brains. (**a**) bEnd.3 cells grown on glass slides were activated with TNFα and IFNγ and incubated with purified αDEC205 or isotype Abs (αGL117) for 30 min. Following washing and fixation, slides were stained with αClaudin 5 Abs followed by TRITC-labeled secondary Abs (red). Bound αDEC205 and αGL117Abs were visualized by incubating with goat-anti-rat AF488 secondary antibody (green). (**b**) Cells were treated as in (**a**) and stained thereafter with LAMP-1 (red) and Claudin 5 (green)-specific Abs and matching secondary reagents. (**c**) WT mice were i.v. injected with 10 µg αDEC205 Abs in 100 µL PBS. After 10 min mice were euthanized and brains were isolated and snap-frozen. Cryosections of the brains were prepared and stained with were stained with goat-anti-rat AF488 secondary Abs (green) to detect in vivo bound DEC205, and αClaudin 5 Abs followed by goat-anti-rabbit TRITC secondary antibody (red). The merged micrograph displays DAPI-stained nuclei in blue.

The regulation of endothelial junctions is a complex process, involving several junctional proteins and signaling events [30]. As Claudin 5 was affected the most by αDEC205 Ab treatment, expression of Claudin 5 proteins by cultivated bEnd.3 cells, using immunofluorescence microscopy, was analyzed next. Staining with Claudin 5 specific Abs revealed a prominent change in distribution and expression when comparing αDEC205 treated ECs with isotype controls (Figure 7a). In controls, Claudin 5 lines up at the cell membranes of ECs, indicating tight cell-to-cell junctions. Upon αDEC205 Ab treatment, Claudin 5 is no longer staining the outline of the ECs, instead it accumulated intracellularly in a perinuclear area. Of interest, Claudin 5 colocalizes with αDEC205 Abs in these intracellular vesicles (Figure 7a). As these vesicles are reminiscent of LAMP-1^+^ compartments that earlier have been shown to be targeted by DEC205 in DCs, double labeling with the lysosomal marker LAMP-1 was performed. Figure 7b shows double labeling of Claudin 5 positive compartments with LAMP-1 after incubation with αDEC205. To confirm these findings in vivo, brain sections were double-stained with Abs against DEC205 and Claudin 5, and analyzed by immunofluorescence microscopy (Figure 7c). Here, double labeling of brain ECs for both antigens was observed. However, a clear colocalization in endocytic vesicles in situ could not be verified due to insufficient resolution of confocal microscopy, but the obvious joint expression of DEC205 and Claudin 5 by brain ECs offers the possibility that αDEC205 Abs destabilize the endothelial junctions by triggering the loss of Claudin 5 from the cell membrane. As a consequence, tight junctions between ECs are weakened and the blood–brain barrier becomes increasingly permeable.

## 4. Discussion

DEC205 has for long been characterized in DCs, but recently expression of DEC205 was also confirmed in neutrophilic granulocytes and B cells [21,22]. Due to its intracellular domain containing a coated pit motive, DEC205 is considered being an antigen uptake receptor [6]. Despite the fact that brain ECs in humans [31] have been shown to be capable of expressing MHC class I and MHC class II molecules, respectively, which is one prerequisite for antigen presentation and T cell activation, direct antigen presentation followed by proliferation of T cells has not been demonstrated yet for brain endothelial cells [32]. As for MHC class I mediated presentation by ECs, this again rather leads to detainment of CD8^+^ T cells from the brain, than to activation. Thus, this leaves an assessory function of MHC expression for ECs. While human ECs have the capacity to present antigens under inflammatory conditions, we could not demonstrate MHC-class II expression by either DEC205^+^ bEnd.3 cells in vitro nor in vivo by brain ECs, which is backed by initial investigations showing that murine ECs are unable to express MHC molecules in the steady state [33]. Thus, a function of DEC205 in antigen uptake and presentation in ECs is rather unlikely.

The endocytic function of DEC205 in ECs may have been modified during evolution depending on the cellular environment, i.e., from antigen uptake, which takes place in antigen-presenting cells (APCs), to transportation of molecules. Specifically, cells lining tissue borders such as ECs and epithelial cells in lung and thymus, need specialized receptors to control uptake of defined molecules and to ensure proper targeting from luminal to abluminal sides. Thus, in contrast to APCs, DEC205 mediated uptake in ECs may help the “en-route” targeting of critical substances by receptor mediated endocytosis. In fact, very early immunohistological investigations with the original αDEC205 Ab clone NLDC-145 may support this transportation function of DEC205, as expression was detected in lung and in thymic epithelial cells [1]. Both cell types are unable to present MHC-class II:peptide complexes to T cells, but need specialized receptors for transportation, as they are part of an epithelial barrier, which prevents unwanted biomolecules from entering the deeper tissues.

For bEnd.3 cells we have shown that engagement of the DEC205 receptor by αDEC205 Abs, used as surrogate ligand, triggers the loss of Claudin 5 from the cell membrane as well as the downregulation of Claudin 5-specific mRNA. This is accompanied by a decrease of the TEER in bEnd.3 monolayers, indicating a loss of the integrity of the endothelial junctions. Similar to DEC205, Claudin 5 is in particular expressed by ECs in the brain and the eye, making it an important part of the blood–brain barrier [34,35]. Expression of Claudin 5 in the cell membranes has been shown to be downregulated by endocytic events triggered by the PTEN/AKT/mTOR pathway [36]. Although DEC205 has not been shown to stimulate signaling events in DCs so far, it nevertheless harbors a casein kinase II sequence in its intracellular domain [4], which may be important to induce signaling events, eventually leading to the uptake of Claudin 5. This notion may be backed by observations in epithelial caco-2 cells, where casein kinase II is recruited to tight junctions regulating Occludin:Claudin 2 interactions by phosphorylation of Occludin [37].

Moreover, the interaction of Claudin 5 with caveolin is proposed to explain the degradational uptake of Claudin 5 and its passage through the endocytic pathways during breakdown of the blood–brain barrier [38,39]. Thus, a similar mechanism of αCD205 triggered co-uptake of Claudin 5 and DEC205 in ECs may be possible, as Claudin 5 itself does not contain a coated pit motive which can enable direct uptake by itself. But as DEC205 contains a coated pit, as well as a distal-targeting motive, it may “chaperone” degradation-bound Claudin 5 through the endocytic pathways [40] to a degrative lysosomal compartment. Although using DEC205 Abs as a surrogate ligand may trigger different signals compared to those triggered by natural ligands, we demonstrate that defined natural ligands, i.e., CpG, as well as different αDEC205 Abs clones, are endocytosed and targeted to intracellular compartments in a manner similar to NLDC145, the αDEC205 clone that was mainly used in this study. This suggests that comparable mechanisms and similar signaling events may be triggered by the uptake of Abs and ligands. Furthermore, the molecular structure of DEC205 lacks ITAM signaling motifs and crosslinking of DEC205 receptor subunits has not been demonstrated. Therefore, polyvalent Abs and monovalent ligands may not differ in their signaling when bound to DEC205.

The exclusive expression of DEC205 by CNS-blood endothelium tempted us to speculate about specific expression by immune privileged sites only [41]. Indeed, ECs in the eye express DEC205, but testis, yet another immune-privileged organ, is negative. Therefore, DEC205 expression by ECs is rather restricted to the brain, and does not seem to be a marker for immune privileged sites in general.

Nevertheless, as DEC205 has frequently been associated with a tolerogenic function of DCs [19], for instance in the eye, they may even be called “veto” cells in lay terms [42]. DEC205 expression by ECs may further contribute to this function [31] as DEC205 may bind antigens similar to DCs, but because ECs miss expression of T cell-activating molecules, such as CD80 and CD86, the presentation of (self-) antigens will avoid T cell activation and may even lead to tolerance by inducing anergy and regulatory T cells [43].

In summary, expression of DEC205 is not only restricted to DCs, neutrophilic granulocytes and B cells, it is also expressed by non-immune cells, i.e., ECs. By executing its function as an endocytosis receptor in ECs, which are non-professional APCs, DEC205 seems to guide the removal of Claudin 5 from the endothelial junctions by regulated endocytosis, and DEC205 can thereby contribute to immune reactions, even when expressed by non-immune cells, as it may weaken the blood–brain barrier, allowing leukocytes to enter the CNS.

## Data Availability

The original contributions presented in this study are included in the article/Supplementary Material. Further inquiries can be directed to the corresponding author.

## Supplementary Materials

The following supporting information can be downloaded at: https://www.mdpi.com/article/10.3390/cells15100882/s1, Figure S1: bEND.3 cells do not function as antigen-presenting cells; Figure S2: DEC205 antibodies target to the perinuclear area of bEND.3 cells; Figure S3: CpG and DEC205 antibodies (clone YECTA) target to lysosomal compartments; Figure S4: bEND.3 cells take up DEC205-PE antibodies after 5 min of pulsing; Figure S5: DEC205 is not expressed by vascular endothelial cells in tissues outside the CNS; Figure S6: Injected αDEC205 antibodies do not target to peripheral endothelial cells; Figure S7: Injection of αDEC205 antibodies promotes extravasation of Evans Blue into the CNS tissues.

## Abbreviations

The following abbreviations are used in this manuscript:

Ab	Antibody
α	Anti-
APCs	Antigen-presenting cells
BMDC	Bone marrow-derived DC
BBB	Blood–brain barrier
CNS	Central nervous system
CRD	Carbohydrate recognition domain
DC	Dendritic cell
EC	Endothelial cell
i.v.	Intravenous
MFI	Mean fluorescence intensity
MIIC	MHC class II compartment
PE	Phycoerythrin
qRT-PCR	Quantitative reverse real-time polymerase chain reaction
TRITC	Tetramethylrhodamine isothiocyanat
TEER	Transendothelial electrical resistance
