# Supplementary material for: The Endocytic Receptor DEC205 Is Expressed by Brain Endothelial Cells and Is Involved in Regulating the Blood–Brain Barrier
Source: Cells. 2026 May 12;15(10):882. doi: 10.3390/cells15100882 (PMC13204628; doi:10.3390/cells15100882)
Supplement: Supplementary file 1 [file cells-15-00882-s001.zip › cells-4190412-supplementary.pdf]

Figure S1

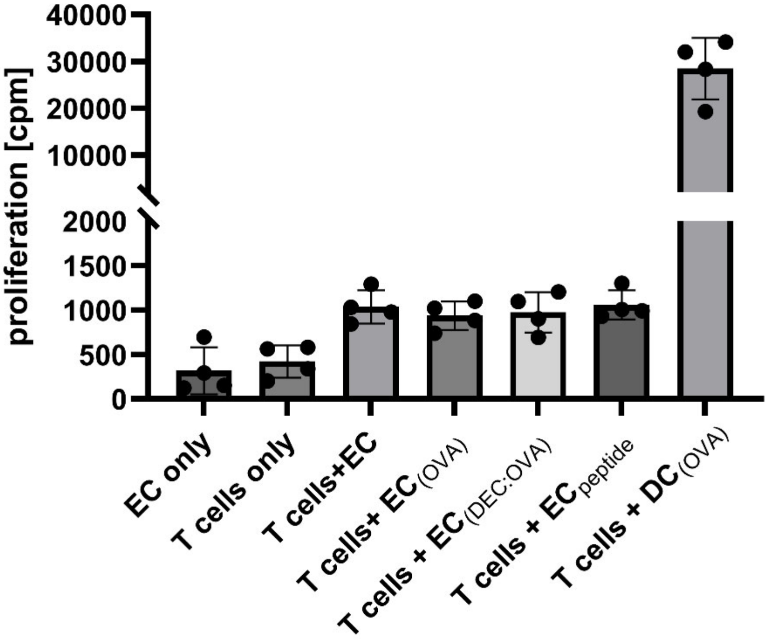

Figure S1. bEnd.3 cells do not function as antigen presenting cells.

Bone marrow derived DCs or bEnd.3 cells were pulsed for 24 h with Ovalbumin (OVA), DEC-conjugated Ovalbumin (DEC:OVA) or with the cognate OVA peptide at 37°C, 5% CO_2. Cells were washed with complete medium and $1x10^4$ cells/well were distributed into 96 well round bottom plates. $CD4^+$ T cells were isolated from OVA-specific DO11.10 mice and $2x10^5$ T cells cells/well were added to the differently pulsed DCs or ECs. Plates were incubated for 3 days and for the last 18 h of culture ^{3}H-Thymidin was added. Cells were harvested and incorporated radioactivity was measured in a beta plate reader (Wallac, Turku, Finland). Only OVA pulsed bone marrow derived DCs induced strong antigen-specific proliferation of the OVA-specific $CD4^+$ T cells.

Figure S2

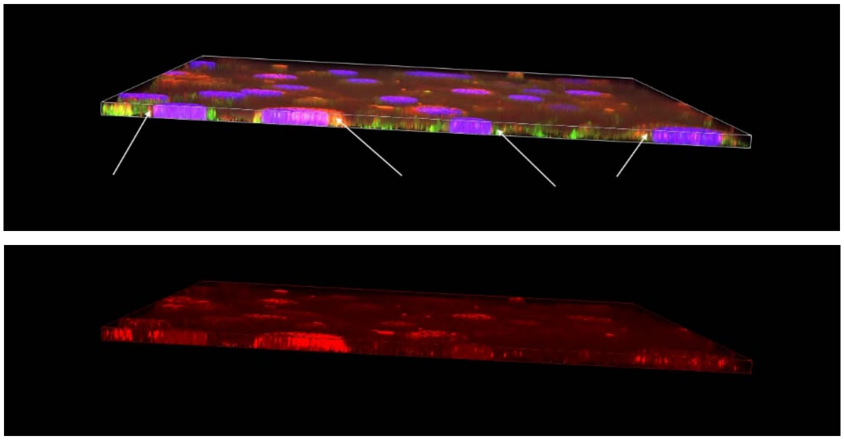

red: DEC205
green: CD144
blue: nuclei

Figure S2. DEC205 antibodies target to the perinuclear area of bEnd.3 cells.
b.End.3 cells were cultivated on glass slides and incubated with DEC205-PE and CD144-Alexa488 (both from BioLegend, Koblenz, Germany) for 30 min at 37°C, 5%CO_2. Thereafter cells were washed, fixed and after staining the nuclei with DAPI, slides were covered with cover slips. Analysis was performed using a Nikon confocal microscope. Optical sections were prepared using NIS elements software (Nikon, Duesseldorf, Germany). Arrows indicate perinuclear labeling of DEC205 inside the cells.

Figure S3

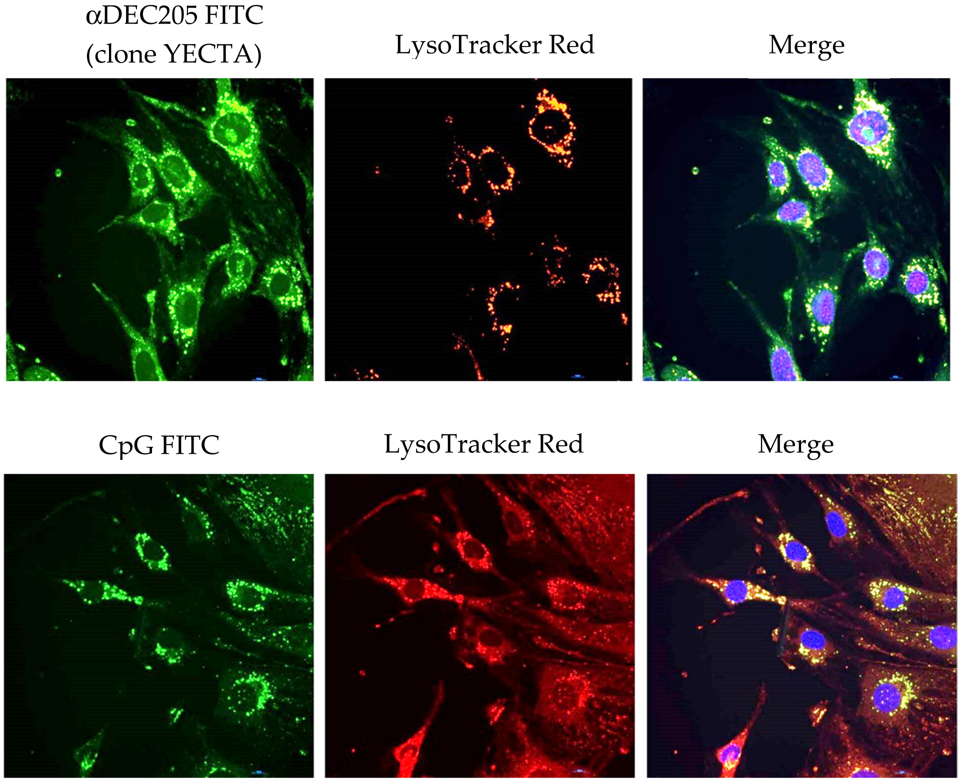

Figure S3. CpG and DEC205 antibodies (clone YECTA) target to lysosomal compartments.
bEnd.3 cells were grown on glass culture slides for 24h. Monolayers of cells were treated with αDEC205-FITC clone YECTA or CpG FITC each together with LysoTracker Red for 30 min at 37°C, 5%CO_2. Thereafter slides were examined by confocal microscopy.

Figure S4

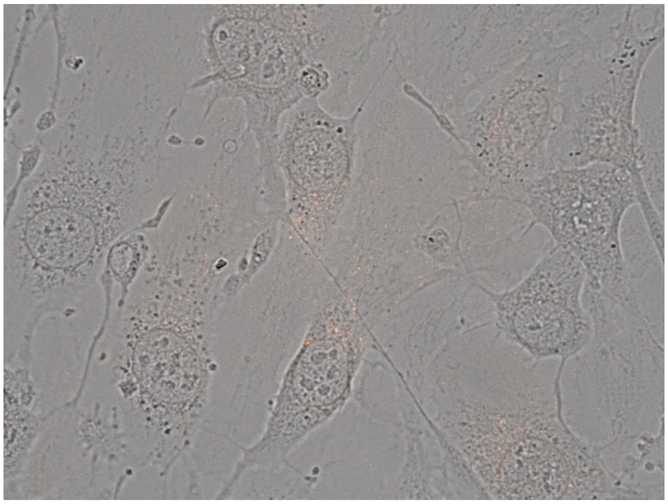

Figure S4. bEnd3 cells take up DEC205-PE antibodies after 5 min of pulsing.

bEnd.3 cells were cultures in ibidi tissue culture slides and incubated with PE-labeled αDEC205 antibodies for 5 min. Thereafter slides were examined by brightfield and immune fluorescence microscopy (Nikon, Duesseldorf, Germany), an overlay is shown. Red dots indicate endocytosed PE-labeled αDEC205 antibodies.

Figure S5

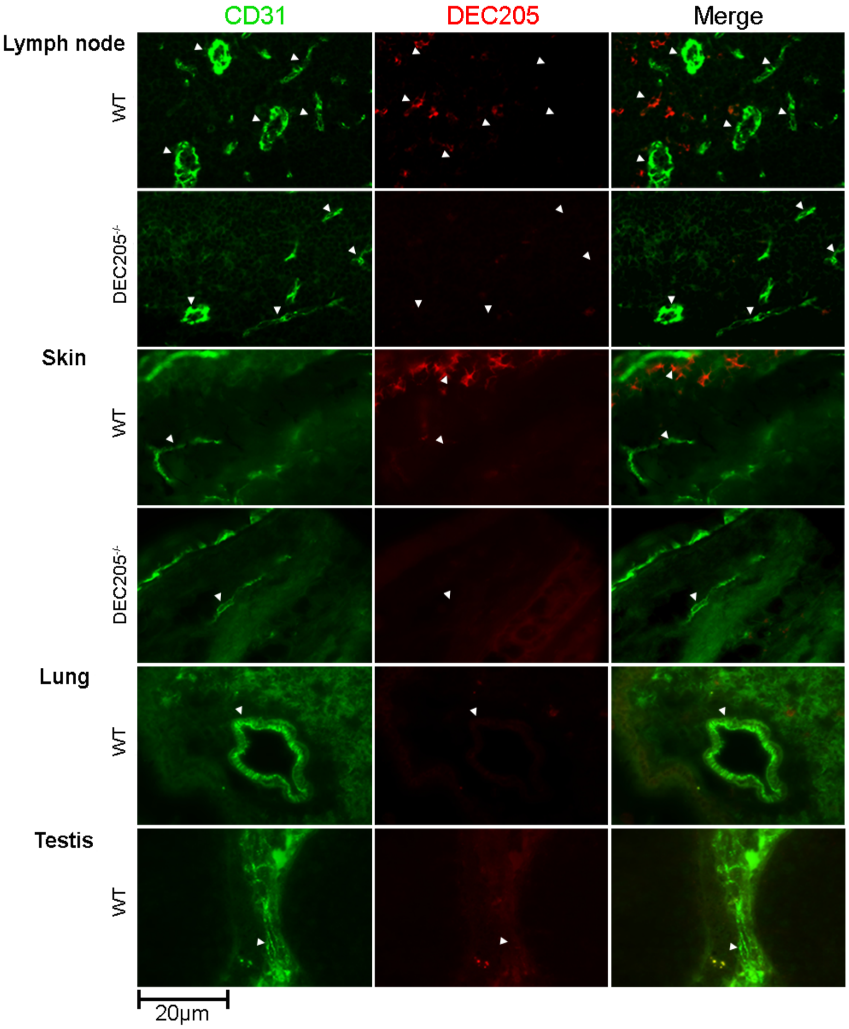
CD31
DEC205
Merge
Lymph node
WT
DEC205-/-
Skin
WT
DEC205-/-
Lung
WT
Testis
WT
20μm

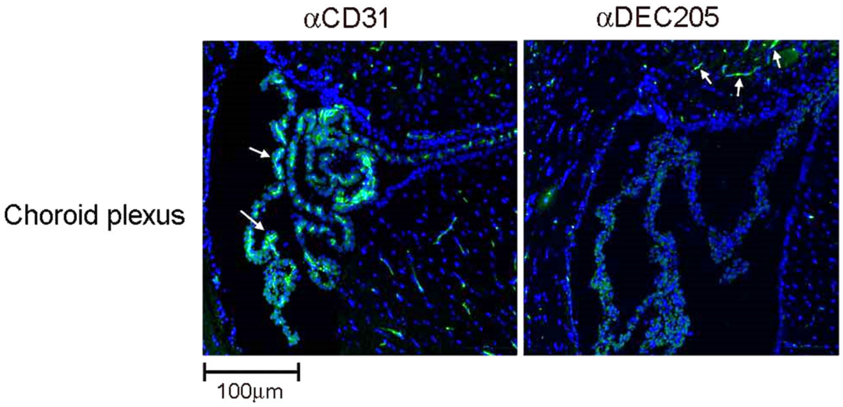
αCD31
αDEC205
Choroid plexus
100μm

Figure S5. DEC205 is not expressed by vascular endothelial cells in tissues outside the CNS.
(a) Cryosections of frozen tissues of lymph node (paracortex), ear (epidermis, dermis), lung and testis (cord) were labeled with αCD31-AF488 and αDEC205-PE Abs. No double-labeling was detected. DEC205 expression is seen in dendritic cells of the WT lymph node section and in the ear expressed by Langerhans cells. Images were captured by immunofluorescence microscopy, and arrows indicate exemplary positive stained vessels and cells. (b) Serial sections of snap frozen brains of WT mice were stained with either αCD31-AF488 or αDEC205-AF488 labeled antibodies and areas with choroid plexus were examined. DEC205 only stained vessels in the surrounding brain area, adjacent to the choroid plexus, whereas all blood vessels in the brain tissue and the choroid plexus were positive for staining with αCD31 Abs.

Figure S6

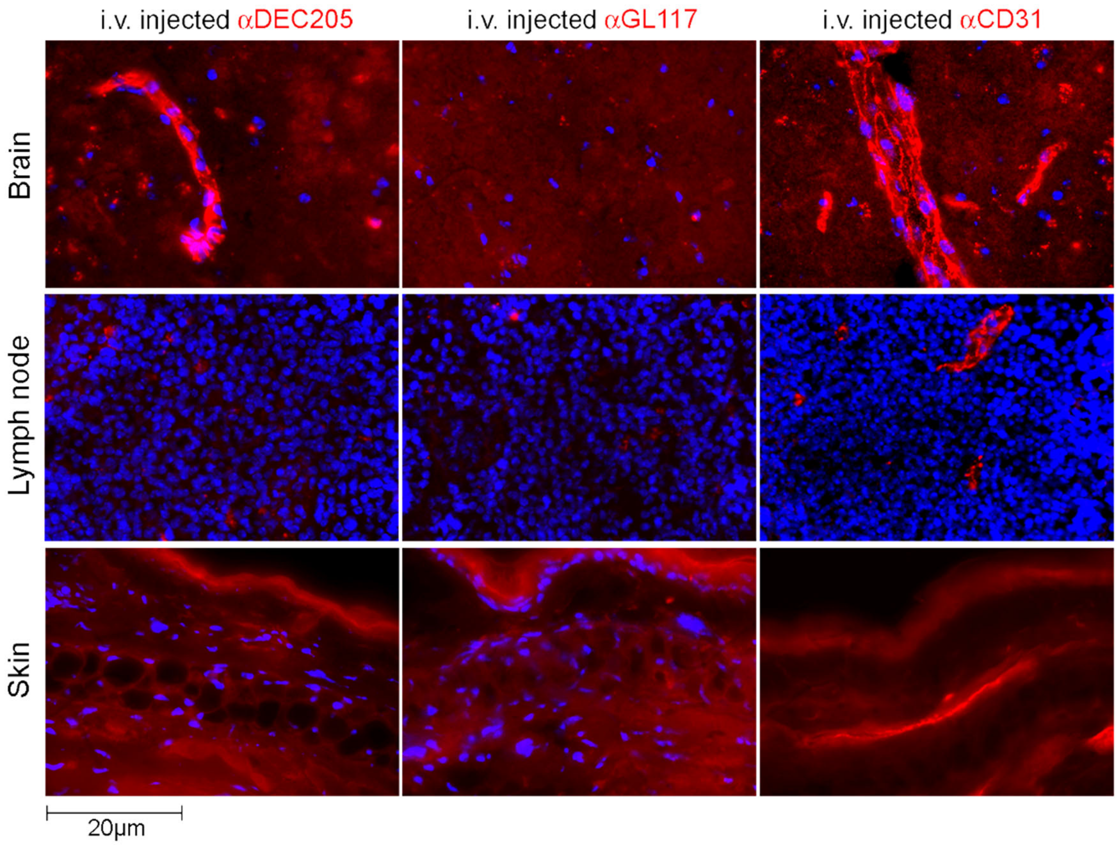

Figure S6. Injected αDEC205 antibodies do not target to peripheral endothelial cells.
αDEC205 or αGL117 Abs (Isotype control) as well as αCD31 (positive control) were i.v. injected into the tail veins of WT mice. After 10 min animals were euthanized and organs as indicated were prepared for cryosectioning. Sections were fixed and stained with the secondary Ab goat-anti-rat PE, to visualize injected and in vivo bound Abs. Sections were examined by immunofluorescence microscopy.

Figure S7

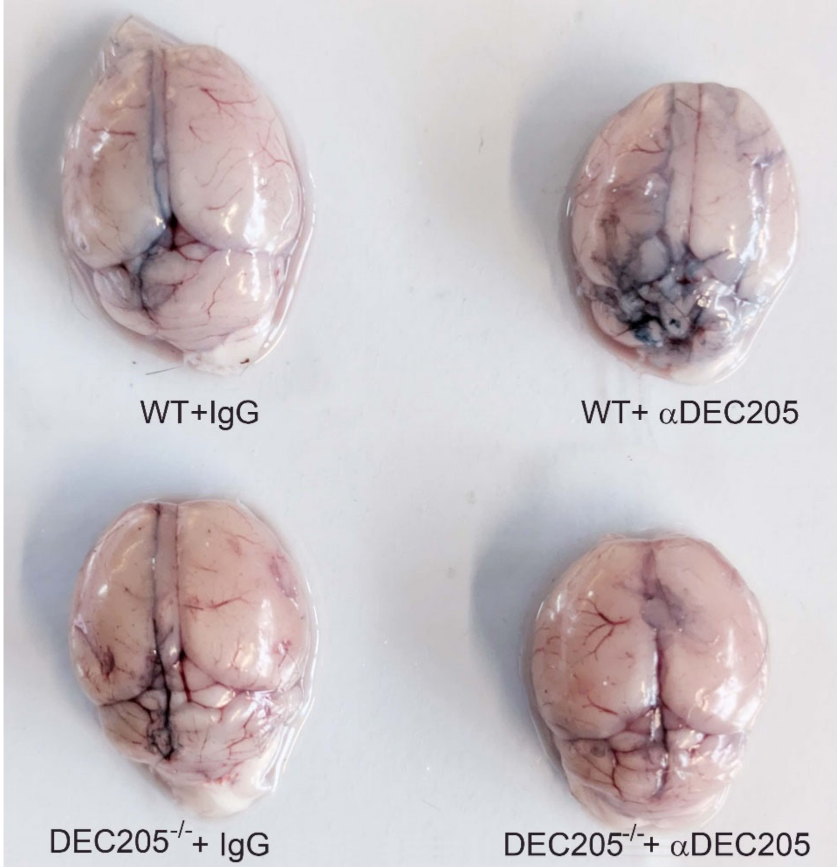

Figure S7. Injection of αDEC205 antibodies promotes extravasation of Evans blue into the CNS tissues. WT mice and DEC205 deficient ($DEC205^{-/-}$) mice were injected with αDEC205 Abs or control IgG (10 μg/ 100 μL PBS per mouse i.v.), followed by i.v. injection of Evans blue (10 mg/mL in PBS; 50 μL/mouse). After 30 min mice were euthanized and brains were prepared and photographed.
